# Modulation of hippocampal gamma oscillations by acetylcholine: insights from mathematical and *in vitro *optogenetic models

**DOI:** 10.1186/1471-2202-16-S1-P267

**Published:** 2015-12-18

**Authors:** Ruth Betterton, Jack Mellor, Krasimira Tsaneva-Atanasova

**Affiliations:** 1 School of Physiology and Pharmacology, University of Bristol, Bristol, BS8 1TD, UK; 2 College of Engineering, Mathematics and Physical Sciences, University of Exeter, Exeter, EX4 4QF, UK

## 

A neuronal oscillation involves the rhythmic, synchronous firing of a population of cells. Oscillations found throughout the cortex can be separated into bands of differing frequencies which are associated with various behavioural states. Gamma oscillations (30 - 100 Hz) occur coincidently with attention, sensory processing and learning and memory. The hippocampus, known for its role in learning and memory, shows gamma activity *in vivo *[1] and gamma oscillations can be induced in *in vitro *slices [2]. The release of acetylcholine (ACh) correlates with increases in oscillatory power *in vivo *[3] and knockout of specific ACh receptor subtypes provides evidence for this scenario [4]. To further investigate the role of ACh in the modulation of gamma oscillations we have utilised both *in vitro *and computational techniques.

We implemented a mathematical model of the CA3 region of the hippocampus based on [5]. Using Hodgkin-Huxley single compartmental neurons, we verify that a network of 80 excitatory pyramidal cells and 20 inhibitory interneurons is able to produce oscillatory activity within the gamma range.

We developed an optogenetic system (see [6,7]) to induce gamma oscillations enabling us to test modulation by specific acetylcholine receptors. Male mice received stereotaxic injection into the CA3 region of the hippocampus of a viral vector (AAV5) containing channelrhodopsin (hChR2(H134R)) under the control of the CaMKIIα promoter. Stimulation of the ChR expressing CA3 pyramidal cell bodies with short light pulses (5-50 ms) evoked action potentials and stimulation of Schaffer collateral axons elicited robust synaptic responses in the CA3 and CA1 regions that were blocked by the application of NBQX (10 µM) or TTX (1 µM). Local field potential recordings showed that a 1s step optical stimulation induced low power and low frequency gamma oscillations which attenuated over time. *In vivo*, gamma oscillations are often found 'nested' within an overlying theta oscillation [8]. Correlating well with these *in vivo *recordings, theta frequency (5 Hz) sine wave optical stimulation induced higher power and higher frequency gamma oscillations with less attenuation over a 1s period.

We introduced, a similar, 5 Hz sine depolarising input to the pyramidal cells in our mathematical model and found that it was able to induce oscillations at gamma frequency.

By manipulating specific currents within the model, we predicted the effect of specific ACh receptor subtype activation on gamma oscillations. These predictions were supported by our *in vitro *experimental evidence showing that we found that activation of ACh receptors did indeed modulate gamma oscillations with M1 receptor having a major effect.
